# Impact of *Helicobacter pylori* eradication on age-specific risk of incident dementia in patients with peptic ulcer disease: a nationwide population-based cohort study

**DOI:** 10.1007/s11357-024-01284-z

**Published:** 2024-08-12

**Authors:** Dong Woo Kang, Jung-Won Lee, Man Young Park, Sung-Hwan Kim, Yoo Hyun Um, Sheng-Min Wang, Chang Uk Lee, Hyun Kook Lim

**Affiliations:** 1 Department of Psychiatry, College of Medicine, The Catholic University of Korea, Seoul St. Mary’s Hospital, Seoul, Republic of Korea; 2 Department of Data Science, Korea Institute of Oriental Medicine, Daejeon, Republic of Korea; 3 Department of Psychiatry, College of Medicine, The Catholic University of Korea, Yeouido St. Mary’s Hospital, 10, 63-Ro, Yeongdeungpo-Gu, Seoul, 06591 Republic of Korea; 4 Department of Psychiatry, College of Medicine, The Catholic University of Korea, St. Vincent’s Hospital, Suwon, Republic of Korea; 5 Research Institute, NEUROPHET Inc, Seoul, Republic of Korea; 6 CMC Institute for Basic Medical Science, The Catholic Medical Center of The Catholic University of Korea, Seoul, Republic of Korea

**Keywords:** Peptic ulcer disease, Helicobacter pylori eradication therapy, Dementia risk, Alzheimer’s disease, Elderly Korean population, Cohort study

## Abstract

The impact of peptic ulcer disease (PUD) and *Helicobacter pylori* (*H. pylori*) eradication therapy on dementia risk in high *H. pylori* prevalence populations remains uncertain. This study investigates the relationship between PUD, *H. pylori* eradication, and dementia risk, including Alzheimer’s disease (AD), in an elderly South Korean cohort, considering age and eradication timing. Data from the Korean National Health Insurance Service (2002–2015) for individuals aged 55–79 were analyzed. Participants were divided based on PUD and *H. pylori* therapy status. Propensity score matching was used to evaluate dementia incidence and hazard ratios over 5 and 10 years, alongside the timing of eradication therapy. PUD is linked to higher dementia risk at 5 and 10 years, more for overall dementia than AD, with eradication status not significantly altering the risk. Age-specific analysis showed increased AD risk in the 60s and 70s age groups. Late eradication therapy is correlated with a higher dementia risk. PUD is a risk factor for dementia in elderly South Koreans, particularly with delayed *H. pylori* therapy. The findings emphasize timely *H. pylori* management and its potential role in neurodegenerative disease prevention.

**Supplementary Information:**

The online version contains supplementary material available at 10.1007/s11357-024-01284-z.

## Introduction

Dementia, which is prevalent in older adults, impairs memory and cognitive functions, affecting independent living. Alzheimer’s disease (AD), the most common form of AD, is mainly explained by the amyloid cascade theory (ACH), which links amyloid beta (Aβ) plaque buildup to neuronal damage and neurofibrillary tangles, causing AD [1]. However, Aβ and tau-related brain changes account for only about half of the cognitive decline over time [2], and Aβ-targeted treatments reduce cognitive deterioration by just 25–30% [3, 4]. This highlights the limitations of ACH and the need to consider other factors, such as inflammation theory. This theory posits that chronic brain inflammation, potentially triggered by infections, not only exacerbates Aβ and tau pathology but also serves as a primary contributor to neurodegeneration, cognitive decline, and dementia [5].

*H. pylori* stands out as a prominent pathogen known to colonize the human stomach mucosa and is notorious for causing peptic ulcer disease (PUD) and gastric cancer [6], with a particularly high prevalence in East Asia [7]. Beyond its effects on the gastrointestinal tract, *H. pylori* has the capability to cross the blood–brain barrier, which contributes to neuroinflammation [8]. This infiltration is believed to be a potential factor in Aβ deposition [9], tauopathy [10], and the increased production of inflammatory cytokines [8]. Additionally, this bacterium plays a role in the production of reactive oxygen metabolites and significantly influences neuronal apoptosis [11]. Furthermore, *H. pylori* infection damages the gastric mucosa, impairing the absorption of essential micronutrients, such as vitamin B12 and iron, and increasing homocysteine levels, which can cause vascular and endothelial damage [12].

A meta-analysis encompassing case–control and cohort studies revealed that *H. pylori* infection increases the risk of all-cause dementia; however, interestingly, it does not elevate the risk of AD [13]. Complementing this, a longitudinal cohort study focusing on older adults further confirmed that *H. pylori* infection increased the overall risk of dementia [14]. However, the outcomes of *H. pylori* eradication treatments are inconsistent. For instance, a follow-up study conducted over 2 years post-eradication treatment observed improvements in cognitive and functional status among patients with AD [15], alongside findings that suggest such treatment may reduce the risk of dementia progression in these patients [16]. However, based on a cross-sectional study, there have also been reports indicating that eradication treatment does not significantly affect the risk of developing AD [17].

Building on the previous discussion regarding the influence of *H. pylori* infection on dementia, it is pertinent to highlight the clinical significance of dementia caused by PUD among *the H. pylori* infection phenotypes. Predominantly, *H. pylori* infection manifests as a mild gastritis phenotype, often asymptomatic, with an ulcer phenotype constituting about 10–15% of cases [18]. Ulcer phenotype is characterized by acid-peptic injuries that cause mucosal breaks that extend to the submucosa [6]. In South Korea, epidemiological data show a notable prevalence of PUD linked to *H. pylori* infection, particularly in older adults [19]. Among the elderly without gastric symptoms, *H. pylori* seroprevalence was approximately 60% in 2005 and 2011, declining slightly to just over 50% in 2016–2017 [19]. The potential for PUD to differentially impact dementia development compared to non-PUD groups arises from its nature as a more advanced *H. pylori* phenotype [6]. The mechanisms linking PUD to an increased risk of dementia may mirror those suggested for the role of *H. pylori* in dementia development. These include exacerbated systemic inflammation, nutritional deficiencies, changes in the microbiome, and subsequent alterations in the gut-brain axis [20, 21]. A population-based study in Taiwan, an East Asian country, demonstrated a higher incidence of PUD in patients with dementia than in the controls [22]. However, other case–control studies have not found that PUD significantly increases dementia risk across age groups or sex post-adjustments [23]. However, these studies were limited by their case–control design and lack of data on *H. pylori* eradication, focusing only on overall dementia or AD risk. Additionally, these analyses generally lacked precise age stratification or, at most, broadly categorized participants as around 80 years old. Furthermore, previous studies evaluating the effect of *H. pylori* eradication on dementia risk did not typically include a control group without a PUD diagnosis, constituting another gap in our understanding of the relationship between PUD, *H. pylori* infection, and dementia.

In this study, utilizing the sample cohort database from The Korean National Health Insurance Service (KNHIS), we focused on elderly groups aged 55–79 years. Our participants were categorized into three distinct groups for analysis: a control group not diagnosed with PUD, a group diagnosed with PUD but who did not receive *H. pylori* eradication treatment, and a group that underwent eradication treatment following PUD diagnosis. This study aimed to assess the risk of overall dementia and dementia due to AD development over 5 and 10 years. To mitigate the potential influence of a history of PUD on dementia risk, our analysis specifically involved older adults who were first diagnosed with PUD between 55 and 79 years of age. Additionally, considering the significant impact of age on dementia onset [24], we further stratified the risk assessment based on baseline age at PUD diagnosis into three categories: 55–59, 60–69, and 70–79 years. Finally, in light of findings indicating the influence of the timing of eradication treatment on gastric cancer risk [25], we also evaluated the risk of dementia development relative to the timing of the commencement of eradication treatment.

## Materials and methods

### Data source

The KNHIS is a mandatory public health insurance system that provides universal coverage to all residents of South Korea [26]. The KNHIS manages vast amounts of data on domestic health insurance subscribers. Among the datasets available to KNHIS is the Sample Cohort Database (DB), which contains health information and medical use records of subscribers from 2002 to 2015. This DB is primarily used to analyze health trends, identify health risks, and assess the effectiveness of health policies. The sample DB was a random sample of 2.2% of all KNHIS subscribers, stratified by age, sex, and region, and organized to represent the entire population. The DB contains a wealth of information, including demographic data, medical records, diagnostic codes, and procedural codes, and can also be used to study a wide range of health-related issues, such as the prevalence of chronic diseases, use of health services, and medical costs.

### Study cohort

#### Study design and patient selection

**Fig. 1:**
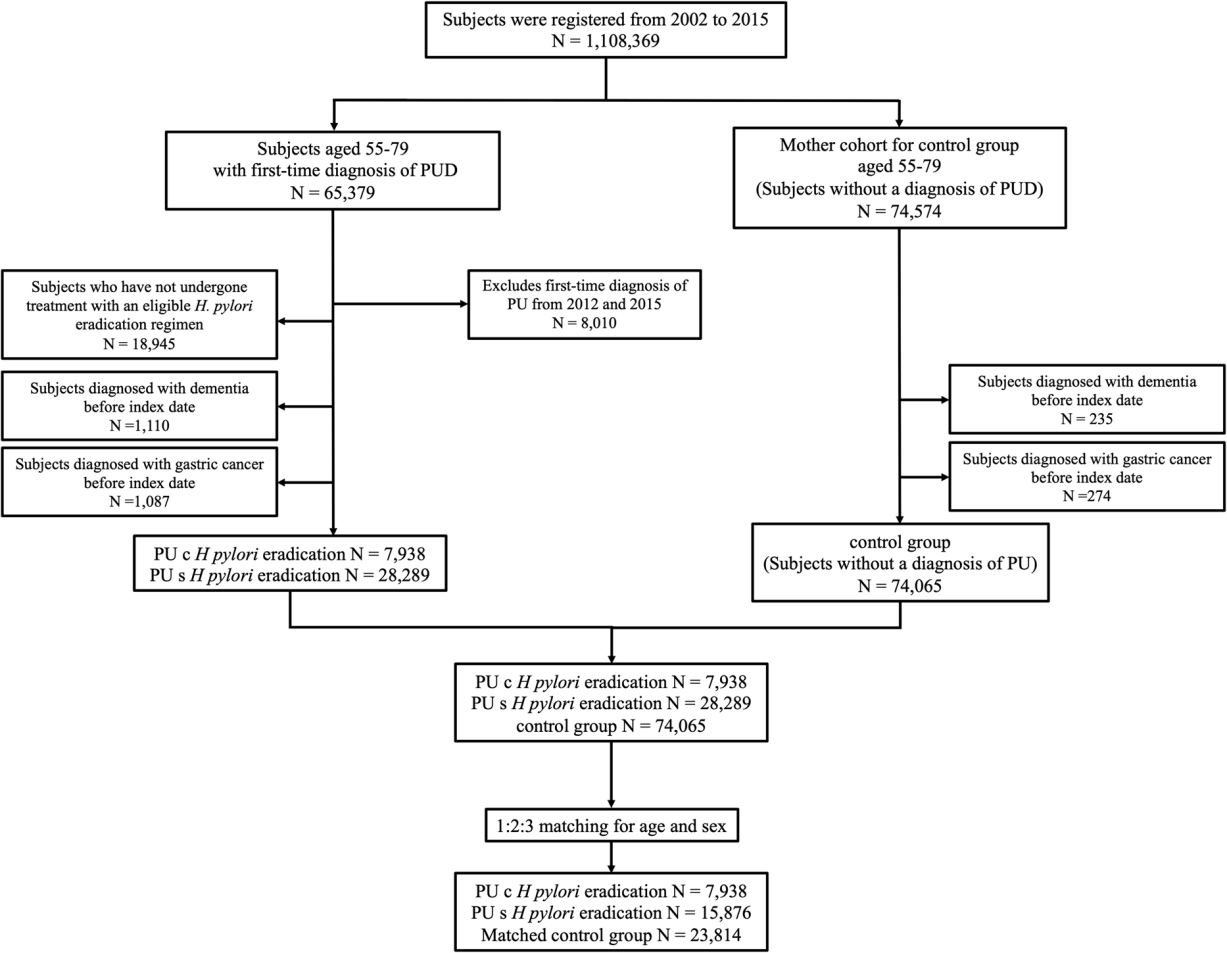
Flow chart depicting creation of study cohorts

Our study was a nationwide, retrospective cohort study employing propensity score matching (PSM) to evaluate the incidence of dementia among patients with PUD, with a specific focus on those who have and have not undergone *H. pylori* eradication therapy. The case- and control-group selection processes are shown in Fig. 1. We initially identified 1,108,369 individuals from the KNHIS National Sample Cohort DB. These participants were divided into those with a first-time diagnosis of PUD (*N* = 65,379) and controls (*N* = 74,574) within the age range of 55–79 years. PUD cases were identified based on the International Classification of Diseases (ICD)-10 codes, K25, K26, and K27. We further narrowed down the PUD group by excluding those diagnosed between 2012 and 2015 to ensure a maximum of 10 years of follow-up for dementia development. A total of 8010 individuals were excluded. Additional exclusion criteria were applied to those who did not receive an appropriate *H. pylori* eradication regimen (*N* = 18,945) and those with prior diagnoses of dementia (ICD-10 codes: G30 or G31, *N* = 1110) or gastric cancer (ICD-10 code: C16, *N* = 1087), leaving 36,227 patients. This cohort was stratified into those who received *H. pylori* eradication therapy after PUD diagnosis (*N* = 7938) and those who did not (*N* = 28,289). The 1-year cutoff was selected based on existing literature indicating a variable impact on gastric cancer risk in patients with PUD [25]. However, to define the clinical significance more clearly in our study, we did not apply the 1-year cutoff directly. Instead, we categorized the patients into early and late *H. pylori* eradication groups based on the timing of initiation of *H. pylori* eradication therapy. The early *H. pylori* eradication group (*N* = 5,580) was defined as the initiation of eradication therapy within 6 months of PUD diagnosis, and the late *H. pylori* eradication group (*N* = 1552) was defined as the initiation of eradication therapy more than 12 months after PUD diagnosis. Details of the eradication regimens, including triple or quadruple therapy, are provided in Supplementary Table 1.

For the control group, after excluding individuals with prior diagnoses of dementia (*N* = 235) or gastric cancer (*N* = 274), 74,065 subjects remained. Controls were age- and sex-matched to case patients using PSM to minimize confounding factors and selection bias, as elaborated in the following sections. The study protocol was approved by the Institutional Review Board of Seoul St. Mary’s Hospital, Seoul, Korea (KC20ZISI1000). Individual consent was not required because publicly available anonymized data were used.

#### Propensity matching

Propensity score adjustment enables the researcher to account for comparability between groups by balancing the distribution of bias and confounders [27]. Thus, it is one of the strongest methods to balance the measured covariates for groups before analysis [28].

We first collected the baseline characteristics of all 7938 subjects from the PUD group with *H. pylori* eradication. Thereafter, propensity was estimated for each participant in the PUD group with *H. pylori* eradication using a multivariate unconditional logistic regression model. Next, using nearest-neighbor matching, each participant in the PUD group with *H. pylori* eradication was paired with a subject from the PUD group without *H. pylori* eradication and controls with the closest propensity score [29]. Age and sex were all included as covariates, and the matching ratio was 1:2:3 for each PUD with *H. pylori* eradication: PUD without *H. pylori* eradication: controls, yielding a total of 23,814 participants in the controls and 15,876 subjects in the PUD group without *H. pylori* eradication. In terms of caliper width, which is the allowable deviation in scores among matches, we set it as 0 for the logit of the propensity score, which was possible because of the large cohort size [30].

#### Outcome variable — incident dementia

In this study, incident dementia was redefined to include both AD and various other primary forms of dementia, collectively referred to as “overall dementia.” This categorization includes dementia diagnoses classified under ICD-10 codes G30 for AD, and G23.1, G31.0, G31.1, G31.82, G31.83, and G31.88 for primary non-AD forms of dementia, as determined by the diagnosis code at the first visit. If both AD and primary non-AD forms of dementia were diagnosed at the first visit, the diagnosis recorded at the second visit was used.

### Statistical analysis

All continuous variables were expressed as mean ± SD and categorical data were presented as numbers (percentages). Study participant characteristics according to PUD diagnosis and *H. pylori* eradication therapy were compared using the analysis of variance (ANOVA) test for continuous variables and the *x*^2^ test for categorical variables. Multivariate Cox proportional hazards regression analysis was performed to identify the 5-year and 10-year hazard ratios (HRs) of overall dementia and dementia due to AD according to the PUD diagnosis with and without *H. pylori* eradication therapy, with controls as a reference category. Model 1 was not adjusted; Model 2 was adjusted for age and sex; Model 3 was adjusted for hypertension, diabetes mellitus, ischemic heart disease, and dyslipidemia; and Model 4 was adjusted for gastric cancer. These variables are known risk factors for incident dementia. The incidence rates (IR) of dementia were calculated by dividing the number of detected cases by the follow-up duration and were expressed as per 1000 person-years for each group. Given the known age-specific risk of incident dementia [24], age-specific IR and HRs were calculated for each age group (55–59, 60–69, and 70–79 years). Finally, in the PU group with *H. pylori* eradication, to determine whether late eradication is an independent prognostic factor for overall dementia and dementia due to AD during the 5- and 10-year follow-up, HRs were calculated using multivariate Cox proportional hazards regression analysis, with the early eradication group as a reference category. The Cox regression model was adjusted for potential confounding factors (Model 1–4). The proportional hazards assumption was tested for all the main effects in all groups. There was no evidence that the proportional hazards assumption was violated in the control and PU groups, with and without *H. pylori* eradication. For all statistical analyses, we used SAS version 9.3 (SAS Institute, Cary, NC, USA), with *P*-values < 0.05 considered significant.

## Results

### Participant characteristics

Supplementary Table S2 shows the baseline characteristics of the study participants before PSM. Before PSM, there were significant differences in age- and age-specific proportions, sex proportions, frequency of peptic ulcer sites, and proportions of hypertension, diabetes, ischemic heart disease, dyslipidemia, and gastric cancer as covariates among the three groups. After PSM, 47,628 participants were included in the study. Among them, 23,814 (50%), 7938 (16.6%), and 15,876 (33.3%) patients were categorized into the control and PUD groups with and without *H. pylori* eradication, respectively. Table 1 summarizes the baseline characteristics of study participants after PSM. There was a significant difference in the proportion of peptic ulcer sites between the PUD groups with and without *H. pylori* eradication. The PUD groups with and without *H. pylori* eradication had the highest proportion of nonspecific peptic ulcers compared to other sites, but the proportion of nonspecific peptic ulcers in the PUD group without *H. pylori* eradication was higher than that in the PUD group with *H. pylori* eradication. Additionally, the proportions of hypertension, diabetes, ischemic heart disease, and dyslipidemia were highest in the PUD group with *H. pylori* eradication. Finally, there was no significant difference among the three groups in the proportion of incident gastric cancer after the index date.

**Table 1:** Baseline characteristics of the study population after propensity score matching

Baseline characteristics	Controls (*N* = 23,814)	Peptic ulcer with *HP* eradication (*N* = 7938)	Peptic ulcer without *HP* eradication (*N* = 15,876)	*P*
Age (mean ± SD)	63.4 ± 6.2	63.4 ± 6.2	63.4 ± 6.2	0.997
- 55–59 [*N* (%)]	7917 (33.2%)	2639 (33.2%)	5278 (33.2%)	1.000
- 60–69 [*N* (%)]	11,454 (48.1%)	3818 (48.1%)	7636 (48.1%)	
- 70–79 [*N* (%)]	4443 (18.7%)	1481 (18.7%)	2962 (18.7%)	
Sex [*N* (% of male)]	12,273 (51.5%)	4091 (51.5%)	8182 (51.5%)	1.000
Peptic ulcer site [*N* (%)]				< .0001
Gastric ulcer	–	2331 (29.4%)	1895 (11.9%)	
Duodenal ulcer	–	1945 (24.5%)	2282 (14.4%)	
﻿ Nonspecific peptic ulcer	–	3662 (46.1%)	11,699 (73.7%)	
Hypertension [*N* (%)]	7 (0.0%)	2272 (28.6%)	1043 (6.6%)	< .0001
Diabetes [*N* (%)]	6 (0.0%)	1618 (20.4%)	778 (4.9%)	< .0001
Ischemic heart disease [*N* (%)]	3 (0.0%)	938 (11.8%)	477 (3.0%)	< .0001
Dyslipidemia [*N* (%)]	11 (0.0%)	2,099 (26.4%)	958 (6.0%)	< .0001
Gastric cancer [*N* (%)]	803 (3.4%)	281 (3.5%)	598 (3.8%)	0.113

HP *Helicobacter pylori*, *SD* standard deviation, *P* value by ANOVA for continuous variables and by *x*^2^ test for categorical variables

### Risk of dementia in patients with peptic ulcer disease according to *Helicobacter pylori* eradication therapy

The IRs and HRs for dementia according to the PUD diagnosis and *H. pylori* eradication therapy are shown in Table 2. For subjects aged 55–79 years, the IR of overall dementia was highest in the PUD group with *H. pylori* eradication throughout the 5-year follow-up (IR = 9.37), followed by the PUD group without eradication (IR = 8.5) and controls (IR = 2.54). In addition, the IR of dementia due to AD was highest in the PUD group without *H. pylori* eradication throughout the 5-year follow-up (IR = 1.38), followed by the PUD group (IR = 1.23) and controls (IR = 0.74). In addition, the 5-year HRs for overall dementia and dementia due to AD were significantly higher in subjects with PU diagnosis, regardless of *H. pylori* eradication, than in controls. After adjusting for covariates in Model 4, the 5-year risk for overall dementia in the PUD group with *H. pylori* eradication was observed as an aHR of 3.85 (95% CI 3.26–4.54, *P* < 0.001), and in the PUD group without it as an aHR of 3.54 (95% CI 3.08–4.07, *P* < 0.001). Similarly, for dementia due to AD, the 5-year risk in the PUD group without *H. pylori* eradication was an aHR of 1.99 (95% CI 1.49–2.65, *P* < 0.001), and in the group with it was an aHR of 1.76 (95% CI 1.2–2.59, *P* < 0.001), post adjustment for Model 4 covariates. Further adjustments using variables in Models 2 through 4 (Table 2) did not change these observations. Regarding the 10-year IR and HRs for overall dementia, the IR in the PUD group with *H. pylori* eradication was 11.95 with an aHR of 2.89 (95% CI 2.59–3.21, *P* < 0.001), and in the group without it, the IR was 11.63 with an aHR of 2.77 (95% CI 2.56–3.01, *P* < 0.001). For dementia due to AD, the IR and HRs in the PUD group without *H. pylori* eradication were 2.14, and an aHR of 1.66 (95% CI 1.4–1.96, *P* < 0.001), respectively, and in the group with it was an IR of 1.84 and an aHR of 1.47 (95% CI 1.15–1.87, *P* < 0.001). The 10-year risk of dementia also remained unchanged even after adjusting for covariates in Models 2–4. However, the 10-year HRs for dementia were lower than the 5-year HRs of dementia (Table 2).

**Table 2:** Risk of dementia in patients with peptic ulcer disease according to *Helicobacter pylori* eradication therapy

	**Group**	**Number**	**Events**	**Duration (Person years)**	**IR per 1000**	**Unadjusted HR (95% CI) (Model 1)**	**Adjusted HR (95% CI) (Model 2)**	**Adjusted HR (95% CI) (Model 3)**	**Adjusted HR (95% CI) (Model 4)**
**(A) 5-year follow-up**									
Overall dementia	Control	23,814	301	118,438	2.54	1 (ref.)	1 (ref.)	1 (ref.)	1 (ref.)
	Peptic ulcer c *HP* eradication	7938	353	37,672	9.37	3.78 (3.24–4.41)	3.87 (3.32–4.52)	3.85 (3.26–4.54)	3.85 (3.26–4.54)
	Peptic ulcer s *HP* eradication	15,876	627	73,779	8.5	3.44 (3–3.95)	3.54 (3.09–4.07)	3.54 (3.08–4.06)	3.54 (3.08–4.07)
Dementia due to Alzheimer’s disease	Control	23,814	88	118,781	0.74	1 (ref.)	1 (ref.)	1 (ref.)	1 (ref.)
	Peptic ulcer c *HP* eradication	7938	47	38,152	1.23	1.7 (1.19–2.43)	1.75 (1.22–2.49)	1.76 (1.2–2.59)	1.76 (1.2–2.59)
	Peptic ulcer s *HP* eradication	15,876	103	74,586	1.38	1.92 (1.44–2.55)	1.99 (1.49–2.64)	1.99 (1.49–2.65)	1.99 (1.49–2.65)
**(B) 10-year follow-up**									
Overall dementia	Control	23,814	1,134	219,669	5.16	1 (ref.)	1 (ref.)	1 (ref.)	1 (ref.)
	Peptic ulcer c *HP* eradication	7938	648	54,218	11.95	2.77 (2.51–3.05)	2.89 (2.62–3.18)	2.88 (2.59–3.21)	2.89 (2.59–3.21)
	Peptic ulcer s *HP* eradication	15,876	1,259	108,288	11.63	2.66 (2.45–2.88)	2.77 (2.56–3.01)	2.77 (2.55–3.01)	2.77 (2.56–3.01)
Dementia due to Alzheimer’s disease	Control	23,814	374	221,901	1.69	1 (ref.)	1 (ref.)	1 (ref.)	1 (ref.)
	Peptic ulcer c *HP* eradication	7938	102	55,532	1.84	1.36 (1.09–1.7)	1.43 (1.15–1.79)	1.46 (1.15–1.87)	1.47 (1.15–1.87)
	Peptic ulcer s *HP* eradication	15,876	237	110,823	2.14	1.55 (1.32–1.83)	1.65 (1.39–1.94)	1.65 (1.4–1.95)	1.66 (1.4–1.96)

Peptic ulcer c *HP* eradication, Peptic ulcer with *Helicobacter pylori* eradication; Peptic ulcer s *HP* eradication, Peptic ulcer without *Helicobacter pylori* eradication; *IR* incidence rate, *HR* hazard ratio, *CI* confidence interval. The results shown are hazard ratios and 95% confidence intervals, with unadjusted HRs (Model 1), HRs adjusted for age and sex (Model 2), additionally adjusted for hypertension, diabetes mellitus, ischemic heart disease, and dyslipidemia (Model 3), and adjusted for Model 3 and gastric cancer (Model 4)

### Age-specific risk of dementia in patients with peptic ulcer disease according to *Helicobacter pylori* eradication therapy

**Fig. 2:**
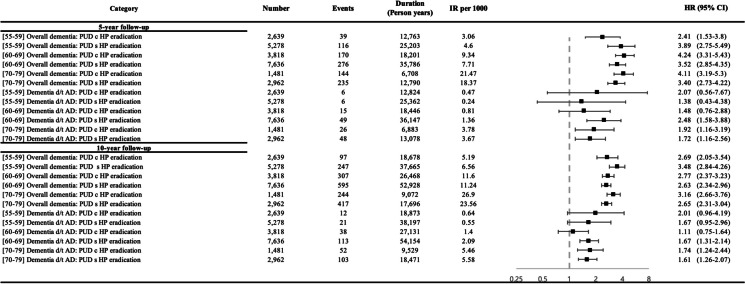
Age-specific risk of dementia in patients with peptic ulcer disease according to *Helicobacter pylori* eradication therapy. PUD c HP eradication, Peptic ulcer disease with *Helicobacter pylori* eradication; PUD s HP eradication, Peptic ulcer disease without *Helicobacter pylori* eradication; IR, incidence rate; HR, hazard ratio; CI, confidence interval. The results shown are hazard ratios and 95% confidence intervals, adjusted for age, sex, hypertension, diabetes mellitus, ischemic heart disease, dyslipidemia, and gastric cancer (Model 4)

Figure 2 shows the age-specific IRs and HRs for dementia according to the PUD diagnosis and *H. pylori* eradication therapy. When stratified by age, in the 55 to 59 years age group, the 5-year risk for overall dementia was observed to be 3.89 (aHR; 95% CI 2.75–5.49, *P* < 0.001) in the PUD group without *H. pylori* eradication, and 2.41 (aHR; 95% CI 1.53–3.8, *P* < 0.001) in the PUD group with *H. pylori* eradication, compared to the controls. In the 60s and 70s age groups, the 5-year risk for overall dementia was 4.24 in the 60 s (aHR; 95% CI 3.31–5.43, *P* < 0.001) and 4.11 in the 70s (aHR; 95% CI 3.19–5.3, *P* < 0.001) in the PUD group with *H. pylori* eradication.

Regarding the 5-year risk for dementia due to AD, there was no significant risk in the PUD group with and without *H. pylori* eradication in subjects aged 55–59 years after adjusting for covariates of Model 4. In subjects aged 60–69 years, we observed an increased risk of dementia due to AD in the PUD group without *H. pylori* eradication compared to controls, after controlling for covariates in Model 4 (aHR 2.48; 95% CI 1.58–3.88, *P* < 0.001). In the age group of 70–79 years, after adjusting for covariates in Model 4, the 5-year risk for AD in the PUD group with *H. pylori* eradication was observed as an aHR of 1.92 (95% CI 1.16–3.19, *P* = 0.01), and in the PUD group without *H. pylori* eradication, an aHR of 1.72 (95% CI 1.16–2.56, *P* = 0.01).

During the 10-year follow-up in the age group of 55 to 59 years, the aHR for overall dementia in the PUD group without *H. pylori* eradication was 3.48 (Model 4, 95% CI 2.84–4.26, *P* < 0.001), in the PUD group with *H. pylori* eradication was 2.69 (Model 4, 95% CI 2.05–3.54, *P* < 0.001), compared to the controls. For those in their 60s with PUD who underwent *H. pylori* eradication, the risk was 2.77 (Model 4, 95% CI 2.37–3.23, *P* < 0.001). For those without eradication treatment, the risk was 2.63 (Model 4, 95% CI 2.34–2.96, *P* < 0.001), compared to the controls. In the 70s age group, the risk was 3.16 (95% CI 2.66–3.76, *P* < 0.001) for PUD patients with eradication treatment, and 2.65 (95% CI 2.31–3.04, *P* < 0.001) for those without it, compared to the controls.

In the 60 to 69 years age group, the 10-year risk for dementia due to AD in the PUD group without *H. pylori* eradication was observed with an aHR of 1.67 (Model 4, 95% CI 1.31–2.14, *P* < 0.01), compared to the controls. In the 70–79 years age group, after adjusting for covariates in Model 4, the 10-year risk for dementia due to AD in the PUD group with *H. pylori* eradication was an aHR of 1.74 (95% CI 1.24–2.44, *P* < 0.001), and in the PUD group without *H. pylori* eradication, the aHR of 1.61 (95% CI 1.26–2.07, *P* < 0.001), compared to the controls.

### Risk of dementia for early and late *Helicobacter pylori* eradication in patients with peptic ulcer disease

In the PUD group with *H. pylori* eradication, we observed a significantly increased 5-year risk for overall dementia and dementia due to AD in the late *H. pylori* eradication group compared with the early eradication group (Model 4, overall dementia: aHR 2.03, 95% CI 1.62–2.53, *P* < 0.001; dementia due to AD: aHR 3.22, 95% CI 1.82–5.69, *P* < 0.001) (Table 3). Additionally, the 10-year risk for overall dementia and dementia due to AD was also higher in the late eradication group than in the early eradication group (Model 4, overall dementia: aHR 1.79, 95% CI 1.19–2.15, *P* < 0.001; dementia due to AD: aHR 2.43, 95% CI 1.57–3.75, *P* < 0.001) (Table 3).﻿

**Table 3:** Risk of dementia for early and late ﻿*Helicobacter pylori* eradication in patients with peptic ulcer disease

	**Group**		**Number**	**Events**	**Duration (Person years)**		**IR per 1000**		**Unadjusted HR (95% CI) (Model 1)**	**Adjusted HR (95% CI) (Model 2)**		**Adjusted HR (95% CI) (Model 3)**	**Adjusted HR (95% CI) (Model 4)**
**(A) 5-year follow-up**													
Overall dementia	Early *HP* eradication	5580		212	26,467		8.01		1 (ref.)	1 (ref.)		1 (ref.)	1 (ref.)
	Late *HP* eradication	1552		127	6525		19.46		2.53 (2.03–3.15)	2.08 (1.66–2.6)		2.02 (1.61–2.52)	2.03 (1.62–2.53)
Dementia due to Alzheimer’s disease	Early *HP* eradication	5580		24	26,766		0.90		1 (ref.)	1 (ref.)		1 (ref.)	1 (ref.)
	Late *HP* eradication	1552		25	6743		3.71		4.42 (2.52–7.75)	3.29 (1.86–5.81)		3.2 (1.81–5.67)	3.22 (1.82–5.69)
**(B) 10-year follow-up**													
Overall dementia	Early *HP* eradication	5580		390	38,061	10.25		1 (ref.)			1 (ref.)	1 (ref.)	1 (ref.)
	Late *HP* eradication	1552		173	8525	20.29		2.12 (1.77–2.54)			1.82 (1.52–2.18)	1.78 (1.48–2.14)	1.79 (1.19–2.15)
Dementia due to Alzheimer’s disease	Early *HP* eradication	5580		54	38,882	1.39		1 (ref.)			1 (ref.)	1 (ref.)	1 (ref.)
	Late *HP* eradication	1552		35	8897	3.93		3.1 (2.03–4.76)			2.45 (1.59–3.78)	2.42 (1.57–3.73)	2.43 (1.57–3.75)

*HP Helicobacter pylori*, *IR* incidence rate, *HR* hazard ratio, *CI* confidence interval. The results shown are hazard ratios and 95% confidence intervals, with unadjusted HRs (Model 1), HRs adjusted for age and sex (Model 2), additionally adjusted for hypertension, diabetes mellitus, ischemic heart disease, and dyslipidemia (Model 3), and adjusted for Model 3 and gastric cancer (Model 4)

## Discussion

In this study, we examined the impact of PUD and *H. pylori* eradication therapy on dementia risk among older adults in South Korea, where the prevalence of *H. pylori* infection is notably high. Our findings indicate a heightened risk of both overall dementia and dementia due to AD associated with PUD over periods of 5 and 10 years. Notably, the risk increase for overall dementia attributed to PUD surpassed that for dementia or AD. The hazard ratio was approximately three times greater in the PUD group than in the control group. Moreover, the *H. pylori* eradication therapy status did not significantly influence the onset of dementia in patients diagnosed with PUD.

Subgroup analyses focusing on age distribution consistently demonstrated an elevated risk of overall dementia within the PUD group across all age brackets. However, no significant differences in dementia risk emerged when stratified according to *H. pylori* eradication therapy status within these age subgroups. In contrast, for dementia or AD, an increased risk was not evident in those diagnosed with PUD aged 55–59, nor in those receiving eradication therapy in their 60s. However, an elevated risk was observed in individuals in their 60s with PUD who did not undergo eradication therapy as well as in those in their 70s with PUD, irrespective of eradication therapy. This pattern suggests that the influence of PUD on dementia risk, particularly d/t AD, may be more significant in specific age groups.

We propose that the effect of PUD and its primary causative factor, *H. pylori* infection, on the onset of dementia may be more closely associated with mechanisms common to overall dementia, rather than AD-specific mechanisms. Notably, *H. pylori* is not neurotropic and its presence in the brains of infected mice has not been documented [31]. We hypothesized that *H. pylori* contributes to the development of dementia through indirect mechanisms, aligned with the inflammatory hypothesis [5]. Chronic inflammation of the gastric mucosa caused by *H. pylori* infection results in increased levels of pro-inflammatory cytokines and astrocytic reactions [10, 32]. In addition, PUD can alter gastric acidity and induce non-*H. pylori* bacterial overgrowth, disrupts gastric flora diversity [33], affects the gut-brain axis, and potentially triggers chronic neuroinflammation and oxidative stress, which are contributors to neurodegeneration [34].

The relationship between PUD and the microbiome is significant as the gastrointestinal tract is home to a diverse microbial community that plays a crucial role in maintaining health and regulating the immune system [35]. Alterations in gastric acidity due to PUD can lead to changes in the microbiome composition, promoting the growth of pathogenic bacteria while reducing beneficial microbial populations [33, 36]. This dysbiosis can affect the gut-brain axis, a bidirectional communication network linking the central nervous system and the gastrointestinal tract, influencing brain function and contributing to neuroinflammation [37].

Furthermore, dementia has been increasingly linked to the microbiome, with several studies suggesting that gut dysbiosis may contribute to neurodegenerative processes [38, 39]. Imbalances in the gut microbiota can result in increased intestinal permeability, allowing microbial metabolites and pro-inflammatory cytokines to enter the bloodstream and reach the brain, potentially triggering or exacerbating neuroinflammation and neurodegeneration [40, 41, 42].

*H. pylori* eradication therapy, while targeting the primary pathogen causing PUD, can also impact the microbiome. The use of antibiotics in eradication therapy can lead to significant and long-lasting disruptions in the gut microbiome, termed gut dysbiosis [43]. This disruption can lead to various metabolic and cardiovascular diseases, as well as potentially influencing dementia risk factors. Antibiotic-induced alterations in the gut microbiota composition have been linked to increased inflammation and changes in metabolic processes, which are risk factors for hypertension, diabetes, ischemic heart disease, and dyslipidemia [44, 45]. Therefore, the observed increase in the prevalence of hypertension, diabetes, ischemic heart disease, and dyslipidemia among patients who underwent eradication therapy for PUD compared to those who did not can be attributed to the impact of antibiotics on the gut microbiome. In this regard, it is essential to consider that while eradication therapy may alleviate the immediate effects of *H. pylori* infection, the broader impact on the microbiome and subsequent health outcomes, including dementia risk, warrants further investigation.

The results of our study suggest that peptic ulcer and *H. pylori* infection might be implicated in dementia risk; however, it is critical to consider asymptomatic *H. pylori* infection. While our study did not directly assess *H. pylori* infection status, epidemiological data indicate a high seroprevalence of *H. pylori* in asymptomatic individuals aged ≥ 50 years in South Korea [19]. Approximately 60% of PUD cases are attributed to *H. pylori* infection [19]. These findings imply that *H. pylori* seroprevalence may be similar regardless of PUD diagnosis. Hence, the impact on dementia risk might not be solely attributable to *H. pylori* infection but also to the impaired regulatory function of acid secretion in the ulcer phenotype [18]. Nevertheless, our study did not directly assess the infection status through serum antibody tests or histological methods, and further research utilizing data that includes infection status is warranted.

Additionally, gastric mucosal damage can lead to the malabsorption of micronutrients, including vitamin B12 and folate. Deficiencies in these nutrients are linked to an increased risk of dementia through various mechanisms including homocysteine elevation, impaired neurotransmitter synthesis, DNA maintenance, and myelin sheath formation [46]. Furthermore, these factors have been reported to promote vascular risk through endothelial damage and oxidative injury, potentially contributing to the overall dementia risk [47].

Our results also imply that the impact of PUD on dementia or AD may be age-specific. An elevated risk was observed primarily in individuals diagnosed with PUD in their 60s who did not undergo eradication therapy and in those diagnosed in their 70 s. Given that neuroinflammation often precedes cognitive impairment and influences its progression [48], and acknowledging the extended duration, often exceeding a decade, required for dementia to manifest in preclinical stages [49], a PUD diagnosis in individuals in their 60s and above, combined with a high concurrent *H. pylori* infection prevalence, could significantly affect dementia risk in later decades. This is particularly pertinent in those in their 70 s and 80 s, who are more vulnerable to dementia. Previous research has shown that *H. pylori* eradication therapy can prompt the regression of precancerous lesions [50], with a notable protective effect against gastric cancer predominantly observed between the ages of 60 and 69 years [25]. This observation aligns with our current findings, in which the risk of dementia due to AD did not show a significant increase in participants who underwent eradication therapy in their 60s. Although direct evidence remains elusive, delaying intestinal metaplasia through eradication therapy could attenuate the impact of *H. pylori* infection and PUD on the risk of developing dementia due to AD [51]. Although our study did not observe age-specific risk variations for overall dementia, these preliminary findings highlight the need for further research. Future studies, particularly those incorporating AD-specific biomarkers and accessible blood biomarkers such as glial fibrillary acidic protein to evaluate the severity of neuroinflammation [52], would be invaluable for determining the effects of eradication therapy on incident dementia.

Prior research indicating an increased risk of gastric cancer in patients initiating *H. pylori* eradication therapy more than 1-year post-PUD diagnosis, as opposed to within a year [25], inspired our hypothesis that the timing of eradication therapy might similarly affect the dementia onset mechanisms linked to PUD and *H. pylori* infection. Our analysis comparing patient groups based on the timing of their eradication therapy (within 6 months of PUD diagnosis versus after 1 year) yielded significant findings: both overall dementia and AD-related dementia incidences were markedly higher in the late *H. pylori* eradication group over a span of 5 and 10 years. Notably, in the PUD cohort, the HR for overall dementia exceeded that for dementia due to AD compared with controls. However, in the late eradication group, the HR for dementia due to AD was higher than that for the overall dementia. This pattern suggests that the timing of eradication therapy may not only be associated with gastric cancer risk but could also potentially influence different types of dementia, although these findings are preliminary. These insights underscore the need for further RCTs to elucidate these complex relationships. Additionally, considering the role of *H. pylori* in increasing the risk of both overall and AD-specific dementia, investigating how the timing of eradication therapy affects AD-related dementia risk is crucial. Assessing changes in AD-specific biomarkers relative to eradication therapy timing could reveal the key underlying mechanisms, offering a more nuanced understanding of the interplay between *H. pylori* infection, eradication timing, and dementia pathogenesis.

Regarding the limitations of our study, several key points must be acknowledged. First, the KNHIS sample cohort database did not include ICD-10 F codes. This omission resulted in the exclusion of vascular dementia cases, primarily caused by vascular lesions in our analysis. While this should be considered when interpreting our findings, it is important to note that vascular risk factors are not necessarily absent in other subtypes of dementia [53]. For instance, approximately 30% of cases primarily diagnosed with AD also present with vascular factors, such as small-vessel disease. Therefore, the influence of vascular risk factors potentially linked to *H. pylori* infection should not be overlooked in the dementia risk assessment of our study. Second, another limitation stems from the nature of the KNHIS database, which lacks direct evidence of *H. pylori* infection and a clear confirmation of successful eradication therapy. Epidemiological studies conducted in South Korea around the time the KNHIS data were collected estimated that approximately 60% of the elderly population, with or without PUD, exhibited *H. pylori* seroprevalence, and the putative eradication rate was around 65% [19]. This context necessitates a cautious interpretation of our findings. Additionally, we acknowledge the importance of patient compliance and the success of eradication therapy in influencing the outcomes. While our current dataset does not include detailed information on these factors, we have explicitly discussed this limitation. We have also highlighted the need for future research to incorporate these crucial variables to better understand their impact on dementia risk.

Lastly, our study did not include information on medications such as acid suppressants and PUD-inducing drugs such as aspirin or NSAIDs, which could be contributing factors to dementia in older adults [54, 55]. Future research should incorporate a comprehensive analysis of these medications to provide a more thorough understanding of the various factors influencing dementia onset.

In conclusion, our study provides preliminary insights into the impact of PUD and *H. pylori* eradication therapy on dementia risk, particularly in the South Korean elderly population. While we observed an increased risk of dementia in patients with PUD, particularly overall dementia, the role of *H. pylori* eradication therapy in modulating this risk remains unclear. Our findings suggest a potential link between *H. pylori* infection, PUD, and dementia, underscoring the complexity of neurodegenerative diseases, and the importance of considering infectious gastrointestinal disorders in their pathogenesis. Future studies should aim to clarify these associations, incorporate more comprehensive data, and explore the mechanisms through which PUD and *H. pylori* might influence dementia development. The nuanced interplay between gastrointestinal and neurological health presents fertile ground for advancing our understanding of dementia, with implications for both prevention and treatment strategies.

## Supplementary Information

Below is the link to the electronic supplementary material.

Supplementary file1 (DOCX 25 KB)

## Data Availability

The data used in this study were sourced from the Sample Cohort Database (DB) of the Korean National Health Insurance Service (KNHIS). Access to the KNHIS Sample Cohort DB is granted following a formal application and approval process via the National Health Insurance Data Sharing Service. Interested researchers can apply for data access at the National Health Insurance Data Sharing Service. Approval is subject to compliance with relevant data protection and privacy regulations as stipulated by the KNHIS.
