# Supplementary material for: Impact of Helicobacter pylori eradication on age-specific risk of incident dementia in patients with peptic ulcer disease: a nationwide population-based cohort study
Source: GeroScience. 2024 Aug 12;47(1):1161–74. doi: 10.1007/s11357-024-01284-z (PMC11872846; doi:10.1007/s11357-024-01284-z)
Supplement: Supplementary file 1 — Supplementary file1 (DOCX 25 KB) [file 11357_2024_1284_MOESM1_ESM.docx]

Supplementary Material

**Impact of *Helicobacter pylori* eradication on age-specific risk of incident dementia in patients with peptic ulcer disease: A nationwide population-based cohort study**

**Dong Woo Kang^1^, Jung-Won Lee^1^, Man Young Park^2^, Sung-Hwan Kim^3^, Yoo Hyun Um^4^, Sheng-Min Wang^3^, Chang Uk Lee^1^, and Hyun Kook Lim^3,5^***

^1^Department of Psychiatry, Seoul St. Mary’s Hospital, College of Medicine, The Catholic University of Korea, Seoul, Republic of Korea

^2^Department of Data Science, Korea institute of oriental medicine, Daejeon, Republic of Korea

^3^Department of Psychiatry, Yeouido St. Mary’s Hospital, College of Medicine, The Catholic University of Korea, Seoul, Republic of Korea

^4^Department of Psychiatry, St. Vincent’s Hospital, College of Medicine, The Catholic University of Korea, Suwon, Republic of Korea

^5^Research Institute, NEUROPHET Inc., Seoul, Republic of Korea

**Running head:** Infections, Cognitive Decline & Dementia

*Address correspondence to Hyun Kook Lim, MD, PhD

Full address: Department of Psychiatry, Yeouido St. Mary’s Hospital, College of Medicine, The Catholic University of Korea, 10, 63-ro, Yeongdeungpo-gu, Seoul, 06591, Republic of Korea; ﻿Tel.: +82 2 3779 1048; Fax: +82 2 780 6577; E-mail: [drblues@catholic.ac.kr](mailto:drblues@catholic.ac.kr)

**Keywords:** Helicobacter pylori, dementia, Alzheimer’s disease, peptic ulcer, cohort study

**Supplementary Methods and materials**

**1. Definition of medical history (covariates)**

﻿ The presence of diabetes mellitus was defined according to the presence of ≥1 claim per year under ICD-10 codes E10-14 and ≥1 claim per year for the prescription of anti-diabetic medication (ATC codes: A10A, A10B, A10X), if this condition was met once during hospitalization or on two or more occasions in outpatient settings. The presence of hypertension was defined according to the presence of ≥1 claim per year under ICD-10 codes I10-15 and ≥1 claim per year for the prescription of antihypertensive agents (ATC codes: C03, C07, C08, C09), if this condition was met once during hospitalization or on two or more occasions in outpatient settings. The presence of ischemic heart disease was defined according to the presence of ≥1 claim per year under ICD-10 codes I20-25. The presence of dyslipidemia was defined according to the presence of ≥1 claim per year under ICD-10 code E78 and ≥1 claim per year for the prescription of a lipid-lowering agent (ATC code: C10). The presence of gastric cancer was defined according to the presence of ≥1 claim per year under ICD-10 code C16.

**Supplementary Table S1. Prescription frequency by type of *Helicobacter pylori* eradication regimen**

| ***Helicobacter pylori* eradication regimens** | **Frequency** |
| --- | --- |
| PPI + clarithromycin+ amoxicillin | 5,114 |
| H2RA + clarithromycin + amoxicillin | 1,018 |
| H2RA + levofloxacin + amoxicillin | 911 |
| H2RA + amoxicillin + metronidazole | 271 |
| PPI+ levofloxacin + amoxicillin | 181 |
| PPI + amoxicillin + metronidazole | 99 |
| H2RA + amoxicillin + bismuth | 78 |
| PPI + clarithromycin + metronidazole | 75 |
| PPI + tetracycline + metronidazole | 52 |
| H2RA + clarithromycin + metronidazole | 42 |
| PPI + amoxicillin + bismuth | 25 |
| PPI + clarithromycin + bismuth | 17 |
| H2RA + tetracycline + amoxicillin | 15 |
| H2RA + tetracycline + metronidazole | 13 |
| H2RA + clarithromycin + bismuth | 7 |
| PPI + metronidazole + bismuth | 6 |
| H2RA + metronidazole + bismuth | 6 |
| PPI + tetracycline + amoxicillin | 3 |
| PPI + clarithromycin + tetracycline | 3 |
| H2RA + clarithromycin + tetracycline | 2 |

﻿NOTE. These drug combinations were prescribed within the same prescription order and the duration of therapy was between 7 and 14 days. PPI, proton pump inhibitor; H2RA, H2-receptor antagonist.

**Supplementary Table S2. Baseline characteristics of the study population after propensity score matching**

| **Baseline characteristics** | **Controls (n=74,065)** | **PUD with *HP* eradication  (n=7,938)** | **PUD without *HP* eradication (n=28,289)** | | | ***p*** |
| --- | --- | --- | --- | --- | --- | --- |
| **Age (mean ± SD)** | 59.9 ± 6.8 | 63.4 ± 6.2 | 65.0 ± 6.7 | \| <.0001 \| \| --- \| | | |
| **- 55-59 [n (%)]** | 47,011 (63.5%) | 2,639 (33.2%) | 7,404 (26.2%) | | <.0001 | |
| **- 60-69 [n (%)]** | 17,341 (23.4%) | 3,818 (48.1%) | 13,044 (46.1%) | |  | |
| **- 70-79 [n (%)]** | 9,713 (13.1%) | 1,481 (18.7%) | 7,841 (27.7%) | |  | |
| **Sex [n (% of Male)]** | 37,500 (50.6%) | 4,091 (51.5%) | 12,381 (43.8%) | | <.0001 | |
| **Peptic ulcer site [n (%)]** |  |  |  | | <.0001 | |
| **﻿Gastric ulcer** | - | 2,331 (29.4%) | 3,297 (11.7%) | |  | |
| **Duodenal ulcer** | - | 1,945 (24.5%) | 4,053 (14.3%) | |  | |
| **﻿ Nonspecific peptic ulcer** | - | 3,662 (46.1%) | 20,939 (74.0%) | |  | |
| **Hypertension [n(%)]** | 210 (0.3%) | 2,272 (28.6%) | 1,766 (6.2%) | | <.0001 | |
| **Diabetes [n(%)]** | 140 (0.2%) | 1,618 (20.4%) | 1,295 (4.6%) | | <.0001 | |
| **Ischemic heart disease [n(%)]** | 74 (0.1%) | 938 (11.8%) | 806 (2.8%) | | <.0001 | |
| **Dyslipidemia [n(%)]** | 186 (0.3%) | 2,099 (26.4%) | 1,565 (5.5%) | | <.0001 | |
| **Gastric cancer [n(%)]** | 1,641 (2.2%) | 281 (3.5%) | 1,086 (3.8%) | | <.0001 | |

HP, ﻿*Helicobacter pylori*; SD, standard deviation; *p* value by ANOVA for continuous variables and by x^2^ test for categorical variables.
